# Chinese Domestic Ducks Evolved from Mallard Duck (*Anas platyrhynchos*) and Spot-Billed Duck (*A. zonorhyncha*)

**DOI:** 10.3390/ani13071156

**Published:** 2023-03-24

**Authors:** Yang Zhang, Qiang Bao, Zhi Cao, Youqing Bian, Yu Zhang, Zhengfeng Cao, Guohong Chen, Qi Xu

**Affiliations:** 1 Key Laboratory for Evaluation and Utilization of Poultry Genetic Resources of Ministry of Agriculture and Rural Affairs, Yangzhou University, Yangzhou 225009, China; 2 Jiangsu Sci-Tech Demonstration Garden of Modern Animal Husbandy, Taizhou 225300, China; 3 Joint International Research Laboratory of Agriculture and Agri-Product Safety, The Ministry of Education of China, Yangzhou University, Yangzhou 225009, China

**Keywords:** duck, mitochondrial DNA, genetic diversity, origin

## Abstract

**Simple Summary:**

Domestic duck populations in China have abundant genetic resources, and this study is the first to evaluate domestic and wild Mallard populations. To explore the genetic diversity and ancestry of duck populations, we analyzed a sequence (619–667 bp) of mitochondrial DNA (mtDNA) in the control region. Two Muscovy duck breeds were included in this analysis, along with wild Mallard ducks, Eastern spot-billed ducks, and 22 domestic duck breeds. Significant genetic diversity was found among domestic duck breeds. These results not only provide valuable insights into the genetic makeup of domestic ducks but also provide clues to the possible origin of domestic ducks in China.

**Abstract:**

China has a rich genetic resource in its 34 domestic duck breeds. In order to detect the genetic diversity and explore the origin of these indigenous duck populations, the mitochondrial DNA (mtDNA) control region was sequenced and analyzed for 208 individual ducks, including 22 domestic breeds, wild Mallards ducks, Eastern spot-billed ducks, White Muscovy ducks, and Black Muscovy ducks. The haplotype diversity (Hd) was 0.653 and the average nucleotide diversity (Pi) was 0.005, indicating moderate genetic diversity. Sixty haplotypes were detected, and the maximum-likelihood (ML) phylogenetic tree and median-joining (MJ) network were generated from the sequence analyses. In this study, haplotypes from the Mallard duck *(Anas platyrhynchos*) were detected in most of the Chinese domestic duck breeds. In addition, the Eastern spot-billed duck (*A. zonorhyncha*) H8 haplotype was detected in two duck breeds. Only two haplotypes were found in Muscovy ducks, suggesting low genetic diversity within this population. The sequence and haplotype analyses revealed that both *A. platyrhynchos* and *A. zonorhyncha* contributed to the evolution of domestic ducks in China.

## 1. Introduction

Waterfowl resources are rich in China, and the Genetic Resources Committee has officially recognized these resources [1,2]. There are many possible origins of indigenous duck breeds in China [2,3,4]. The ducks may have originated from the domestication of the wild Mallard ducks (*Anas platyrhynchos*), the interbreeding of domesticated Mallard ducks and domesticated Eastern spot-billed ducks (*A. zonorhyncha*), or the domestication of wild hybrids of *A. platyrhynchos* and *Anas zonorhyncha* [3]. Previous studies of genetic diversity using amplified fragment length polymorphism (AFLP) of genomic DNA or analyses of genetic distance and relationships determined that domestic duck breeds evolved from both *A. platyrhynchos* and *A. zonorhyncha* [5,6,7]. Using microsatellite markers of genomic DNA polymorphisms, Chen et al. determined that the contribution to domestic duck breeds was greater for *A. platyrhynchos* than for *A. zonorhyncha* [8]. He et al. also used microsatellite DNA markers from 449 domestic ducks and wild Mallard ducks to demonstrate that all domestic duck breeds in China evolved from a single origin [9]. Similarly, Sultana et al. analyzed six Southeast Asian duck populations and determined that the ducks were domesticated from *A. platyrhynchos* [10]. Overall, these studies support the evolution of Chinese domestic ducks from both *A. platyrhynchos* and *A. zonorhyncha*. 

It was confirmed that the mitochondrial DNA (mtDNA) could more effectively evaluate the genetic diversity of poultry [11]. Unlike nuclear DNA, mtDNA acquires higher mutation rates that are 10 times than those of nuclear genomic DNA, and it is generally not recombined with the mtDNA of the parents [12]. Previous studies have confirmed that the control region has a higher mutation rate compared to the coding region [13]. In addition, it is estimated that mitochondrial control regions have a 2–5 fold higher evolution rate than mitochondrial protein-coding genes, making them more suitable for studying genetic variation among breeds [14]. In addition, a previous study using partial sequencing of mtDNA showed that nine domestic duck breeds along the Yangtze River evolved solely from *A. platyrhynchos* [15]. However, the major limitation of these genetic diversity studies was the limited number of domestic duck breeds and, more specifically, the lack of seven important duck breeds identified by the state, including the Fengtou duck (FT), Mawang duck (MW), Taiwan duck (TW), Zhongshan duck (ZS), Zongyang duck (ZY), White Muscovy duck (BF), and Black Muscovy duck (HF). 

This study performed the first comprehensive evaluation of domestic duck populations and wild Mallard ducks in China. We analyzed a 619–667 bp sequence of the control region of the mitochondrial DNA (mtDNA) of 22 domestic duck breeds, in addition to wild Mallard ducks, Eastern spot-billed ducks, and two Muscovy duck breeds. Sequence and haplotype analyses of the region revealed a high genetic diversity among the domestic duck breeds evaluated in this study. In addition, the identification of haplotypes shared across duck species suggested that Chinese domestic ducks originated from *A. platyrhynchos* and *A. zonorhyncha*.

## 2. Materials and Methods

### 2.1. Ethics Approval

All animal experiments were performed in accordance with the Regulations for the Administration of Experimental Animals issued by the Ministry of Science and Technology (Beijing, China). All experiments were approved by the Animal Care and Use Committee of Yangzhou University (approval code: 151-2014.).

### 2.2. Collection of Blood Samples and DNA Extraction

**Figure 1:**
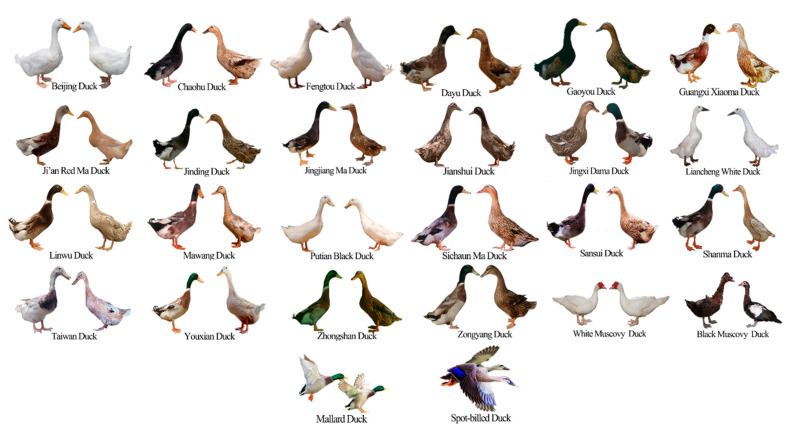
Images of 26 individual duck populations. Images were captured with a digital camera (Olympus, Japan). High-resolution images of each representative duck population are shown in Supplementary Figure S1.

According to the pedigree records, 208 blood samples were collected from unrelated duck individuals (Table 1, Figure 1). In addition, two Muscovy duck populations, the White Muscovy duck (*n* = 8) and the Black Muscovy duck (*n* = 8), were collected from Putian in Fujian Province and used as the reference group for the analysis. These blood samples (approximately 3 mL) were collected from the wing vein of the ducks in individual vacuum blood collection tubes. The genomic DNA was extracted using a genomic DNA extraction kit (TIANGEN, Beijing, China). RNA was purified to remove genomic DNA (gDNA), miRNA, and rRNA using the RNeasy Micro kit (Qiagen, Hilden, Germany), RNase-Free DNase kit (Qiagen), and RiboZero™ MagneticKit (Epicentre, Illumina, San Diego, CA, USA), respectively. Data collection and analysis were conducted at the College of Animal Science and Technology, Yangzhou University, Yangzhou, Jiangsu Province, China.

### 2.3. Polymerase Chain Reaction (PCR) and DNA Sequencing

**Figure 2:**
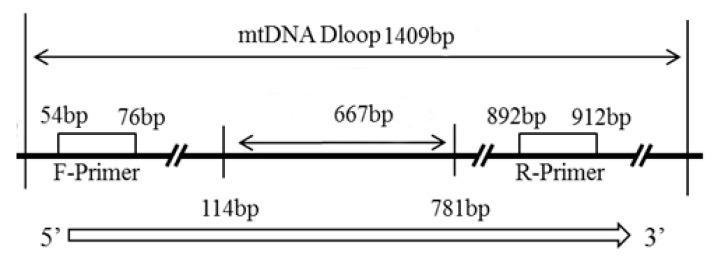
The site of primers according to the Beijing duck sequence (GenBank accession number NC_009684L16770).

The 667-bp (Mallard duck) or 619-bp (Muscovy duck) segment of the mtDNA control region was PCR-amplified using the primers 5′-CCTATGGTCCCGGTAATAAACA-3′ (F-Primer) and 5′-GATAACGCAGGTGTGTCCAG-3′ (R-Primer), which were designed according to the Beijing duck sequence (accession number NC_009684) in GenBank (Figure 2). The PCR assay was conducted in a 20 μL reaction mixture containing 2 μL of buffer (10×), 2.5 μL of MgCl_2_ (25 mM), 1.0 μL of dNTPs (10 mM), 1 μL of each primer (10 μM), 0.2 μL of Taq DNA polymerase (5 U/μL) (Takara Biomedical Technology (Beijing) Co., Ltd.), 1.0 μL of DNA template (100 ng), and of 11.3 μL of sterilized water. The reaction was brought to a final volume of 20 μL by adding double-distilled autoclaved water. PCR amplification was carried out with an Eppendorf Mastercycler (Eppendorf, Hamburg, Germany). The thermocycler was set as follows: 95 °C (5 min), 94 °C (45 s), 58 °C (45 s), 72 °C (45 s) for 35 cycles, and a final elongation step at 72 °C for 5 min. The last elongation step was extended to 8 min at 72 °C, and samples were held at 4 °C. The F-Primer and R-Primer were provided by Sangon Biotech Shanghai Co., Ltd. (Shanghai, China), for the direct bidirectional sequencing of target fragments within the PCR products using the ABI 3730 XL sequencing platform.

### 2.4. Data Analysis

Sequences of the mtDNA fragments were visualized by Chromas2 software and manually browsed to ensure the accuracy of the bases. Sequences were aligned by DNAStar-MegAlign V.7.10. The genetic diversity parameters of the populations, such as variable nucleotide sites, haplotype diversity, and nucleotide diversity, were calculated by DnaSP V.5.0 software [16,17]. The haplotype ratio was calculated as the ratio of the number of haplotypes to the sample size. The distances (Kimura 2-parameter) among all populations were calculated using Mega v.5.0 [18], and then the maximum-likelihood(ML) evolutionary tree was mapped out. Finally, the median-joining (MJ) network of the control region of the mtDNA haplotypes was drawn using Popart v.1.7 software [19].

## 3. Results

### 3.1. Nucleotide Composition of the mtDNA Region of 26 Duck Populations

The data of 208 ducks were analyzed, and the average composition of the sequences was 25.01% A, 15.71% G, 34.23% C, and 25.05% T (50.06% A + T, 49.94% G + C), showing little base bias. A total of 99 polymorphic sites (10 singleton variable sites and 89 parsimony-informative sites) were detected. As all insertions and deletions were removed from the analysis, the variable types were classified as transitions and transversions.

### 3.2. Genetic Diversity of 26 Duck Populations in China

**Figure 3:**
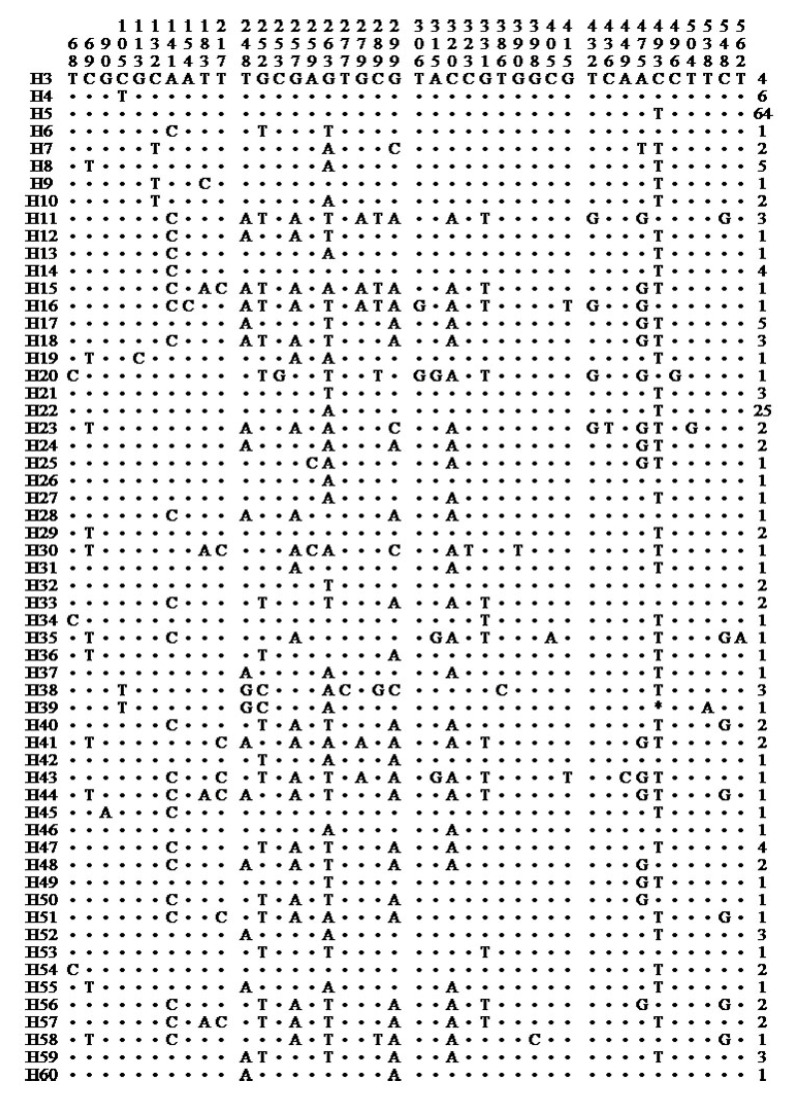
Variable sites in the mtDNA control region for haplotypes from domestic and Mallard ducks. **·** indicates identical nucleotides. Numbers on far right indicate the number of individual ducks with the haplotype.

A total of 60 haplotypes were identified among 26 duck breeds (Table S1), including 2 haplotypes of Muscovy ducks (Table 2) and 58 haplotypes (Figure 3) of domestic ducks, wild ducks, and spot-billed ducks. The largest number of haplotypes (six) were detected in the Chaohu duck (CH), Ji’an Red Ma duck (JA), Jingxi Dama duck (JX), and Zongyang duck (ZY), with a haplotype proportion of 75.0%, while the lowest haplotype proportion (12.5%) was detected in the Sichuan Ma duck (SC) population (Table 3).

The average haplotype diversity (Hd), nucleotide difference (K), and nucleotide diversity (Pi) in these 26 duck populations were 0.653, 3.104, and 0.005, respectively; the Zhongshan duck (ZS) had the highest (Hd = 0.929, K = 6.893, Pi = 0.0111), while Sichuan Ma duck (SC) and White Muscovy duck (BF) had the lowest (Hd = 0.000, K = 0.000, Pi = 0.000). 

### 3.3. Phylogenetic Analysis of Chinese Duck Populations

**Figure 4:**
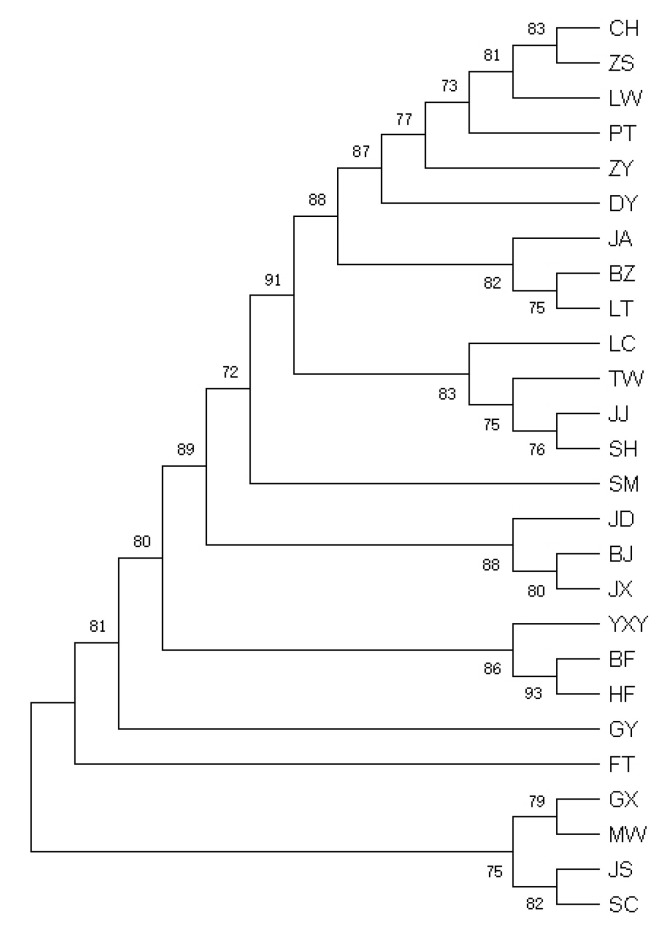
Phylogenetic tree based on mtDNA control region sequences for the 26 duck populations.

**Figure 5:**
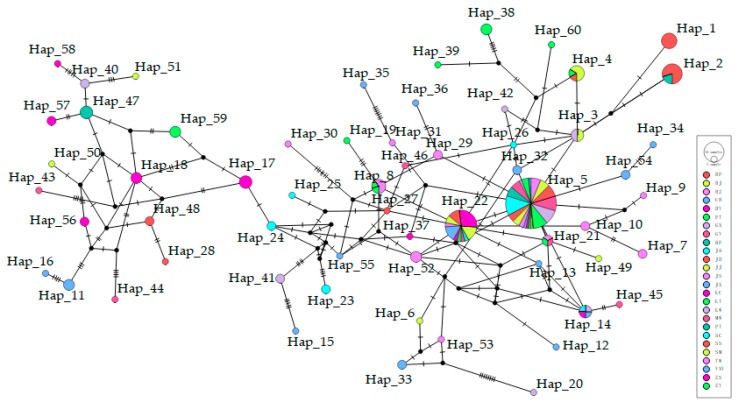
The median-joining networks of mtDNA D-loop haplotypes. The colors represent different duck populations.

The major H5 haplotype was shared by 61 ducks (30.77% of all ducks) from 18 domestic breeds and 3 wild Mallard ducks. The H8 haplotype was shared by one Mallard duck, one Zongyang duck, and one spot-billed duck (Table 3). The ML phylogenetic tree (Figure 4) of the 26 Chinese duck populations and the median-joining network chart (Figure 5) was constructed using all the mallards (*A. platyrhynchos*) and spot-billed ducks (*A. zonorhyncha*) in this study.

## 4. Discussion

Studies on the origin and evolution of livestock breeds through the different mtDNA sequences also contribute to the understanding of the domestication process of species [20]. For the identification of the genetic diversity and phylogeny of different breeds and populations, mtDNA control region sequence analysis has proven to be the most valuable tool [21]. Some studies support the finding that natural selection affects the genetic diversity of populations, which can be measured by haplotype diversity (Hd) and nucleotide diversity (Pi) [22,23,24]. It has been shown that evaluating the impact of natural selection on genetic diversity through haplotype diversity (Hd) and nucleotide diversity (Pi) can help us better understand natural selection and the trends in population genetic diversity trends. In this study, the Hd and Pi of 26 Chinese domestic duck populations were determined to be 0.653 and 0.005, respectively. These results indicate that the domestic duck populations have a relatively large number of haplotypes and exhibit fewer nucleotide sequence differences. In addition, these values were slightly higher than the detected diversity values of four domestic duck breeds in Fujian Province (Hd = 0.645, Pi = 0.001) [25], but they were much lower than those of *A. platyrhynchos* (Hd = 0.987 and Pi = 0.008 in Kulikova’s study) [26]. In comparison with other animals, the Pi of Chinese ducks was higher than that of chickens (<0.001) [27,28,29] and swine (0.001) [30], but it was significantly lower than that of cattle (0.021) [31]. Haplotype and nucleotide diversity were highest in ZS ducks and lowest in SC ducks and BF Muscovy ducks.

Studies on the genetic diversity and genetic structure of species are important components of biodiversity conservation [32]. Previous studies have also found that the SC and BF duck breeds may have experienced a long duration of high selective pressure for particular traits, resulting in low genetic diversity [33,34,35]. By contrast, the ZS population was more recently discovered and was bred in closed regions (Guangdong Province) without any artificial selection. Interestingly, the higher selection pressure might have led to some genotypes being better suited to specific environments [34]. Therefore, this selection increased the frequency of certain genotypes in the population, thereby reducing genetic diversity and even leading to the extinction of some genotypes. In addition, the between-population analyses showed a lower average nucleotide difference (K) than the within-population analyses in this study. Previous studies have found low genetic diversity across 24 domestic Chinese duck breeds and concluded that efficient measures should be adopted to protect domestic duck resources, especially for the JX and LW breeds [15,35]. The results presented here suggest the SC and BF breeds require more immediate attention because of their low genetic diversity.

However, there is still no consensus in the field regarding the origin of domestic ducks in China [2,36]. In this study, the 192 domestic Chinese ducks presented 58 haplotypes, while the 16 Muscovy duck individuals conformed to two haplotypes. The H5 haplotype was shared by the majority of domestic and Mallard ducks, suggesting that most domestic duck breeds in China originated from *A. platyrhynchos*. The Eastern spot-billed ducks clustered into the H7–H10 haplotypes. Interestingly, the H8 haplotype was shared by one Mallard duck, one ZY duck, and one spot-billed duck, indicating that the partial domestic duck breeds in China might have originated from *A. zonorhyncha.* However, there were no other shared haplotypes between the domestic and spot-billed duck populations. Finally, our results suggested that domestic Chinese ducks evolved from *A. platyrhynchos* and *A. zonorhyncha*.

## 5. Conclusions

Sixty haplotypes were detected in most of the domestic Chinese duck breeds analyzed in this study. The sequence and haplotype analyses revealed that both *A. platyrhynchos* and *A. zonorhyncha* contributed to the evolution of domestic ducks in China. The Sichuan Ma duck (SC) and White Muscovy duck (BF) had the lowest Hd, K, and Pi. To summarize, their conservation requires more attention and prioritization of conservation funds in the future.

## Figures and Tables

**Table 1:** Characteristics of the 26 duck populations.

Breed (Abbrev)	Sample Size	Breeding Flock	Conservation Type and Economic Use	Sample Source
Beijing (BJ)	8	450 (50♂, 400♀)	CF (M)	Beijing Golden Star Co., LTD (Beijing, China)
Chaohu (CH)	8	400 (50♂, 350♀)	CF (M & E)	Anhui Academy of Agricultural Sciences (Anhui, China)
Dayu (DY)	8	500 (50♂, 450♀)	CF (M)	Dayu Duck Conservation Farm (Jiangxi, China)
Fengtou (FT)	8	500 (50♂, 450♀)	CF (O)	Zhenjiang Waterfowl Institute (Jiangsu, China)
Gaoyou duck (GY)	8	450 (50♂, 400♀)	CF (E & M)	National Waterfowl Preservation Field (Jiangsu, China)
Guangxi Xiaoma (GX)	8	450 (50♂, 400♀)	CF (E & M)	National Waterfowl Preservation Field (Jiangsu, China)
Ji’an Red Ma (JA)	8	500 (50♂, 450♀)	CF (M & E)	Jiangxi Agricultural University (Jiangxi, China)
Jinding (JD)	8	450 (50♂, 400♀)	CF (E)	National Waterfowl Preservation Field (Fujian, China)
Jingjiang Ma (JJ)	8	450 (50♂, 400♀)	CF (E)	National Waterfowl Preservation Field (Jiangsu, China)
Jianshui (JS)	8	450 (50♂, 400♀)	CF (M)	National Waterfowl Preservation Field (Fujian, China)
Jingxi Dama (JX)	8	450 (50♂, 400♀)	CF (M &E)	National Waterfowl Preservation Field (Jiangsu, China)
Liancheng White (LC)	8	450 (50♂, 400♀)	CF (E & M)	National Waterfowl Preservation Field (Fujian, China)
Linwu (LW)	8	450 (50♂, 400♀)	CF (M & E)	National Waterfowl Preservation Field (Jiangsu, China)
Mawang (MW)	8	450 (50♂, 400♀)	CF (E)	National Waterfowl Preservation Field (Jiangsu, China)
Putian Black (PT)	8	450 (50♂, 400♀)	CF/Z (E)	National Waterfowl Preservation Field (Fujian, China)
Sichuan Ma (SC)	8	350 (50♂, 300♀)	CF (M & E)	Sichuan Agricultural University (Sichuan, China)
Sansui (SS)	8	450 (50♂, 400♀)	CF (E & M)	National Waterfowl Preservation Field (Fujian, China)
Shan Ma (SM)	8	450 (50♂, 400♀)	CF (E)	National Waterfowl Preservation Field (Fujian, China)
Taiwan (TW)	8	450 (50♂, 400♀)	CF (E & M)	National Waterfowl Preservation Field (Fujian, China)
Youxian (YX)	8	450 (50♂, 400♀)	CF (E)	National Waterfowl Preservation Field (Jiangsu, China)
Zhongshan (ZS)	8	450 (50♂, 400♀)	CF (M & E)	National Waterfowl Preservation Field (Fujian, China)
Zongyang (ZY)	8	400 (50♂, 350♀)	CF (M & E)	Anhui Academy of Agricultural Sciences (Anhui, China)
Mallard (LT)	8	350 (50♂, 300♀)	CF (O)	Wuxi Zoo (Jiangsu, China)
Spot-billed (BZ)	8	350 (50♂, 300♀)	CF (O)	Wuxi Zoo (Jiangsu, China)
White Muscovy (BF)	8	450 (50♂, 400♀)	CF (M)	National Waterfowl Preservation Field (Fujian, China)
Black Muscovy (HF)	8	450 (50♂, 400♀)	CF (M)	National Waterfowl Preservation Field (Fujian, China)

Note: CF = conservation farm; M = meat, E = egg, O = ornamental.

**Table 2:** Variable sites in mtDNA for haplotypes of Muscovy ducks.

Haplotype	Nucleotide Site		Total Number of Ducks with the Haplotype
	187	458	
H1	G	T	10
H2	A	C	6

**Table 3:** Haplotype diversity (Hd), average number of differences (K), and nucleotide diversity (Pi) of the control region of mtDNA in 26 duck populations.

Breed (Abbrev)	Sample Size	Number of Haplotypes	Haplotypes (%)	Hd	K	Pi
Beijing (BJ)	8	3	37.5	0.750	8.143	0.001
Chaohu (CH)	8	6	75.0	0.893	1.607	0.002
Dayu (DY)	8	2	25.0	0.536	3.500	0.005
Fengtou (FT)	8	3	37.5	0.464	1.500	0.002
Gaoyou (GY)	8	3	37.5	0.464	3.500	0.005
Guangxi Xiaoma (GX)	8	3	37.5	0.464	0.464	0.001
Ji’an Red Ma (JA)	8	6	75.0	0.929	5.500	0.009
Jinding (JD)	8	4	50.0	0.643	2.357	0.003
Jingjiang Ma (JJ)	8	2	25.0	0.571	0.571	0.001
Jianshui (JS)	8	4	50.0	0.750	3.143	0.005
Jingxi Dama (JX)	8	6	75.0	0.929	3.143	0.005
Liancheng White (LC)	8	2	25.0	0.250	0.500	0.001
Linwu (LW)	8	5	62.5	0.893	6.929	0.011
Mawang (MW)	8	5	62.5	0.786	6.571	0.011
Putian Black (PT)	8	2	25.0	0.571	3.429	0.005
Sichuan Ma (SC)	8	1	12.5	0.000	0.000	0.000
Sansui (SS)	8	5	62.5	0.857	3.643	0.006
Shan Ma (SM)	8	4	50.0	0.750	3.750	0.006
Taiwan (TW)	8	5	62.5	0.857	2.000	0.003
Youxian (YX)	8	4	50.0	0.750	1.714	0.003
Zhongshan (ZS)	8	4	50.0	0.929	6.893	0.011
Zongyang (ZY)	8	6	75.0	0.893	3.429	0.005
Mallard (LT)	8	4	50.0	0.786	5.143	0.008
Spot-billed (BZ)	8	4	50.0	0.821	2.429	0.004
White Muscovy (BF)	8	2	25.0	0.000	0.000	0.000
Black Muscovy (HF)	8	2	25.0	0.429	0.857	0.001

## Data Availability

All data generated or analyzed during this study are included in this published paper.

## Supplementary Materials

The following supporting information can be downloaded at: https://www.mdpi.com/article/10.3390/ani13071156/s1, Figure S1: High-resolution images of each representative duck population; Table S1: The proportion of individuals with each haplotype in duck populations.
