# Supplementary material for: Chinese Domestic Ducks Evolved from Mallard Duck (Anas platyrhynchos) and Spot-Billed Duck (A. zonorhyncha)
Source: Animals (Basel). 2023 Mar 24;13(7):1156. doi: 10.3390/ani13071156 (PMC10093112; doi:10.3390/ani13071156)
Supplement: Supplementary file 1 [file animals-13-01156-s001.zip › Table S1.pdf]

Table S1. The proportion of individuals with each haplotype in duck populations.

Breed (abbrev)	Haplotype	Proportion
Beijing duck (BJ)	H3-H6	25%, 50%, 12.5%, 12.5%
Chaohu duck (CH)	H1-H16	37.5%, 12.5%, 12.5%, 12.5%, 12.5%, 12.5%
Dayu duck (DY)	H17-H18	62.5%, 37.5%
Fengtou duck (FT)	H4- H5, H19	12.5%, 75%, 12.5%
Gaoyou duck (GY)	H5, H21-H22	75%, 12.5%, 12.5%
Guangxi Xiaoma duck (GX)	H5, H14, H20	75%, 12.5%, 12.5%
Ji'an Red Ma duck (JA)	H14 , H22-H26	12.5%, 12.5%, 25%, 25%, 12.5%, 12.5%
Jinding duck (JD)	H4-H5, H27-H28	12.5%, 62.5%, 12.5%, 12.5%
Jingjiang Ma duck (JJ)	H5, H22	50%, 50%
Jianshui duck (JS)	H5, H29-H31	50%, 25%, 12.5%, 12.5%
Jingxi Dama duck (JX)	H5, H32-H36	12.5%, 25%, 25%, 12.5%, 12.5%, 12.5%
Liancheng White duck (LC)	H22, H37	87.5%, 12.5%
Linwu duck (LW)	H3, H5, H40-H42	25%, 12.5%, 25%, 25%, 12.5%
Mawang duck (MW)	H5, H43-H46	50%, 12.5%, 12.5%, 12.5%, 12.5%
Putian Black duck (PT)	H5, H47	50%, 50%
Sichuan Ma duck (SC)	H5	100%
Sansui duck (SS)	H5, H22, H48	37.5%, 37.5%, 25%
Shan Ma duck (SM)	H5, H22, H49-H51	37.5%, 25%, 12.5%, 12.5%, 12.5%
Taiwan duck (TW)	H5, H21-H22, H52-H53	25%, 12.5%, 12.5%, 37.5%, 12.5%
Youxian duck (YX)	H5, H22, H54-H55	12.5%, 25%, 50%, 12.5%
Zhongshan duck (ZS)	H5, H14, H22, H56-H58	12.5%, 12.5%, 12.5%, 25%, 25%, 12.5%
Zongyang duck (ZY)	H5, H8, H21-H22, H59-H60	12.5%, 12.5%, 12.5%, 12.5%, 37.5%, 12.5%
Mallard duck (LT)	H5, H8, H38-H39	37.5%, 12.5%, 37.5%, 12.5%
Spot-billed duck (BZ)	H7-H10	25%, 37.5%, 12.5%, 25%
White Muscovy duck (BF)	H2	100%
Black Muscovy duck (HF)	H1-H2	75%, 25%
