# Supplementary figures and images for: Chinese Domestic Ducks Evolved from Mallard Duck (Anas platyrhynchos) and Spot-Billed Duck (A. zonorhyncha)
Source: Animals (Basel). 2023 Mar 24;13(7):1156. doi: 10.3390/ani13071156 (PMC10093112; doi:10.3390/ani13071156)

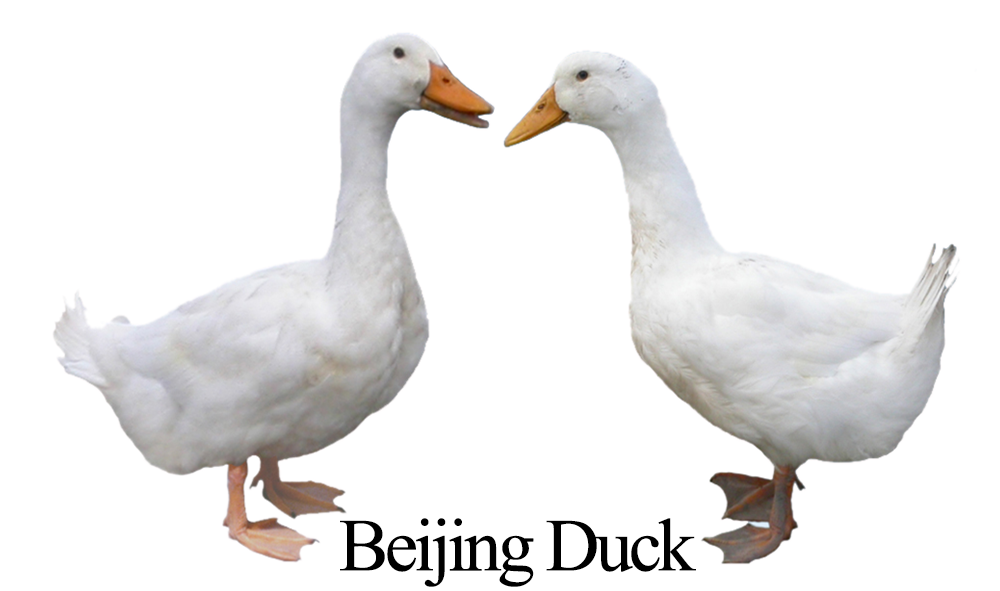

Supplement: Supplementary file 1 [file animals-13-01156-s001.zip › Figure S1/Beijing Duck.tif]

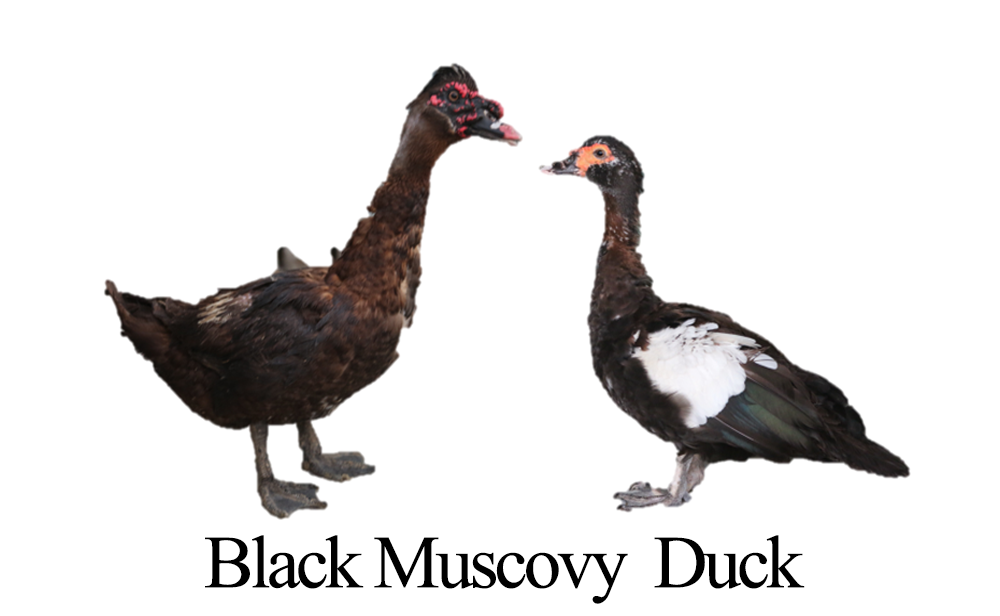

Supplement: Supplementary file 1 [file animals-13-01156-s001.zip › Figure S1/Black Muscovy Duck.tif]

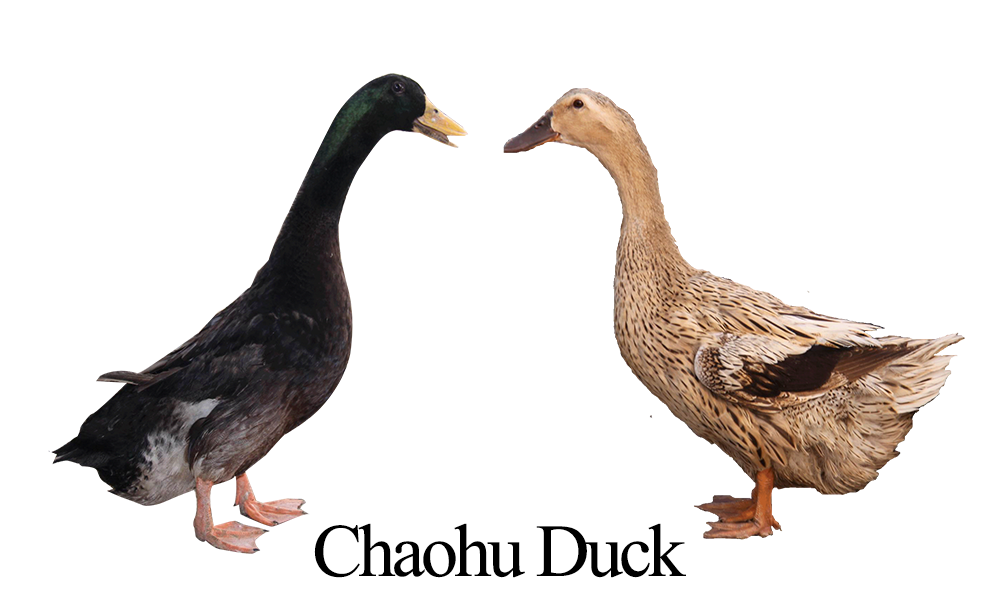

Supplement: Supplementary file 1 [file animals-13-01156-s001.zip › Figure S1/Chaohu Duck.tif]

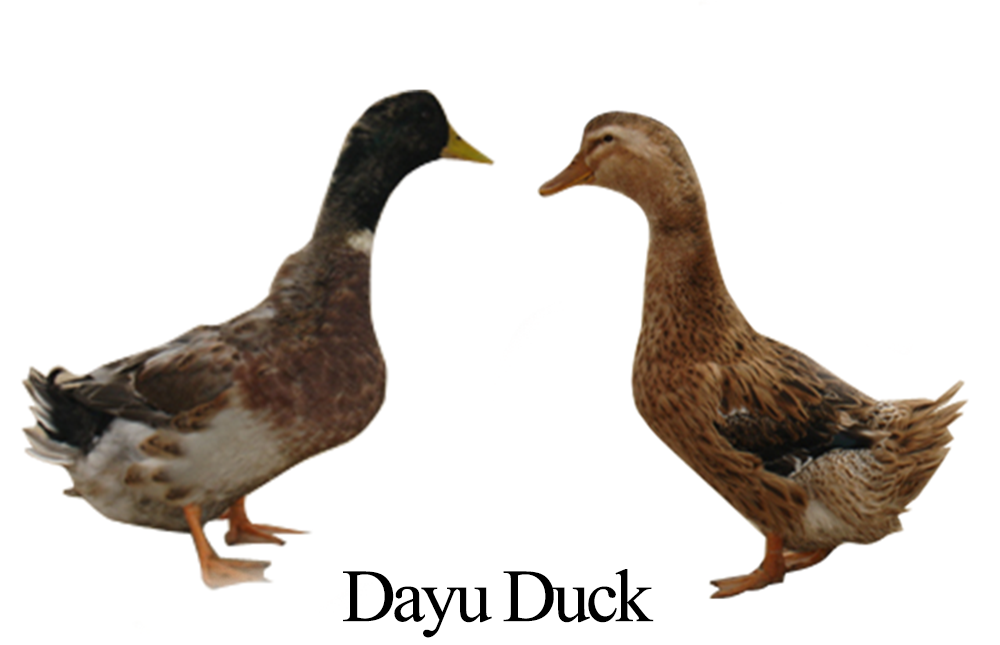

Supplement: Supplementary file 1 [file animals-13-01156-s001.zip › Figure S1/Dayu Duck.tif]

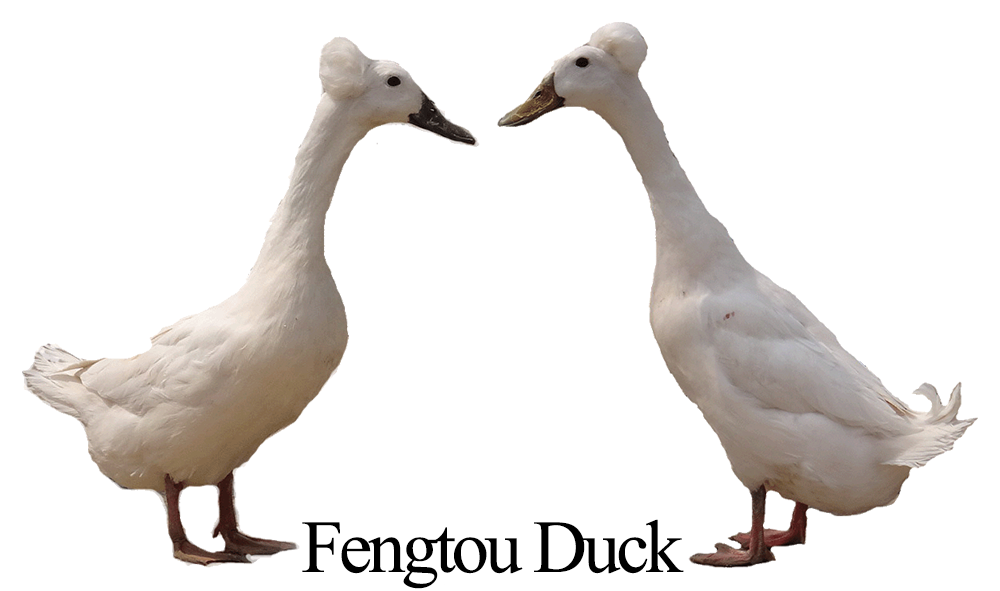

Supplement: Supplementary file 1 [file animals-13-01156-s001.zip › Figure S1/Fengtou Duck.tif]

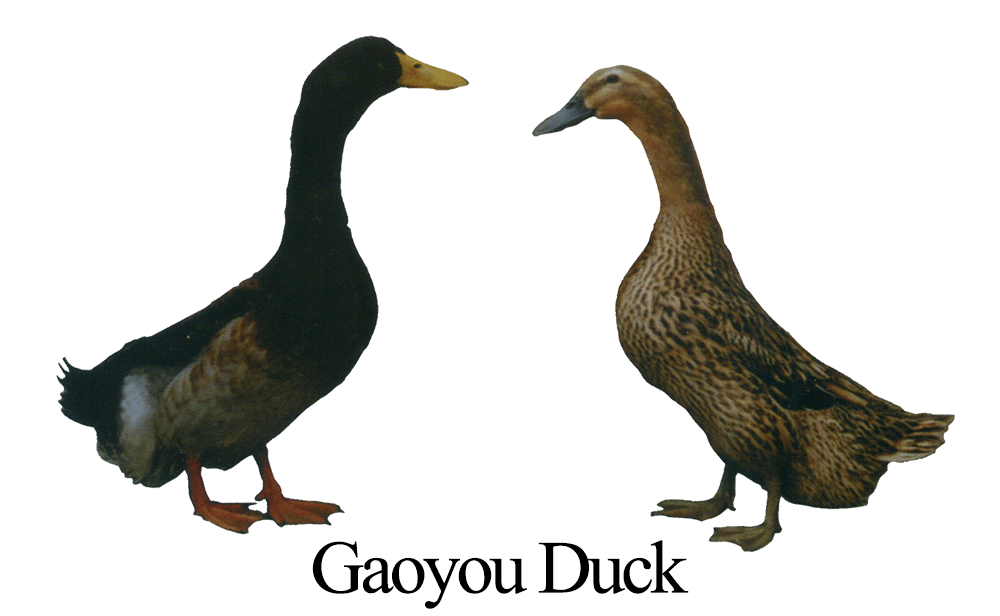

Supplement: Supplementary file 1 [file animals-13-01156-s001.zip › Figure S1/Gaoyou Duck.tif]

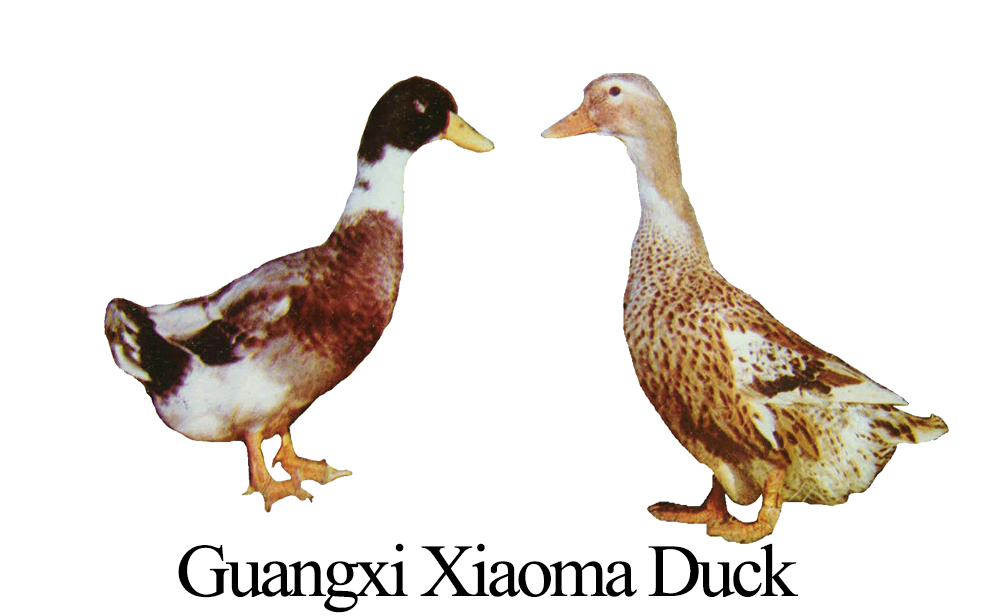

Supplement: Supplementary file 1 [file animals-13-01156-s001.zip › Figure S1/Guangxi Xiaoma Duck.tif]

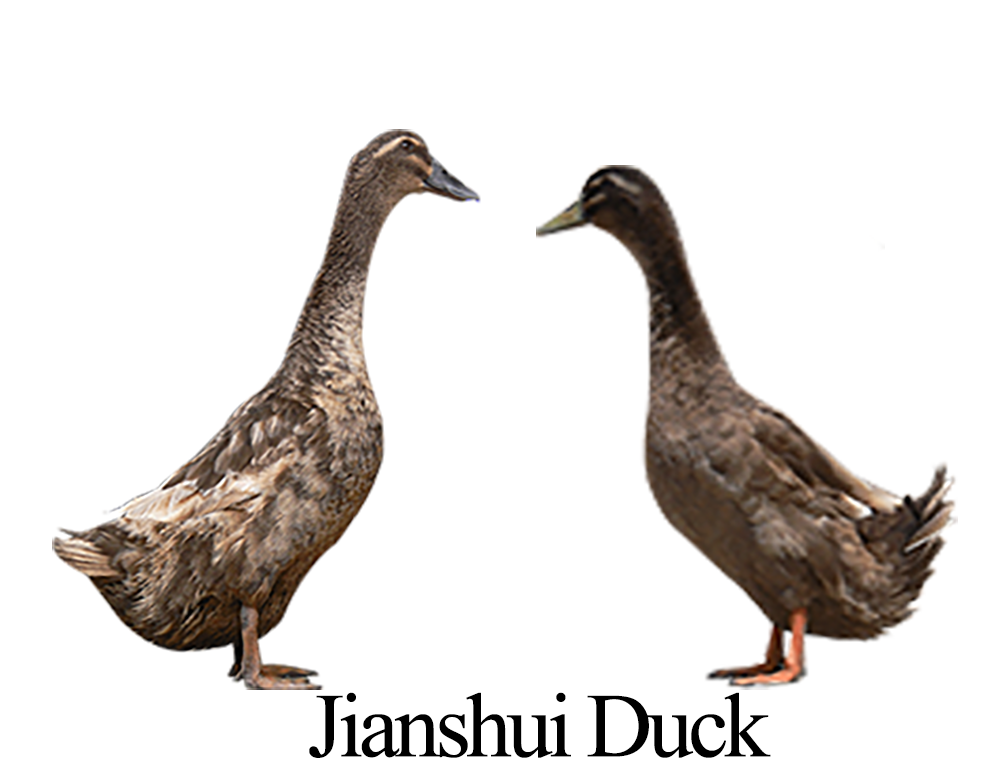

Supplement: Supplementary file 1 [file animals-13-01156-s001.zip › Figure S1/Jianshui Duck.tif]

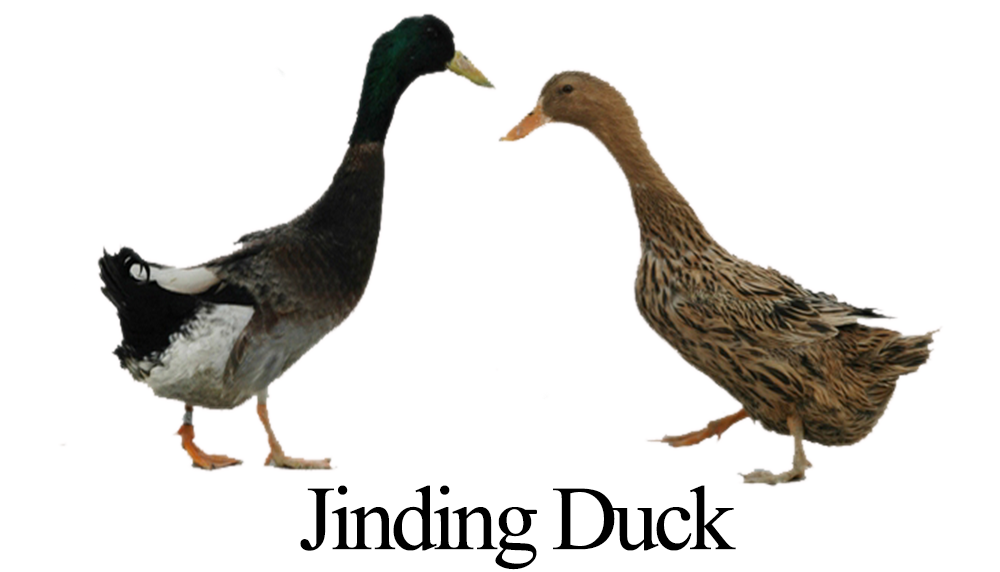

Supplement: Supplementary file 1 [file animals-13-01156-s001.zip › Figure S1/Jinding Duck.tif]

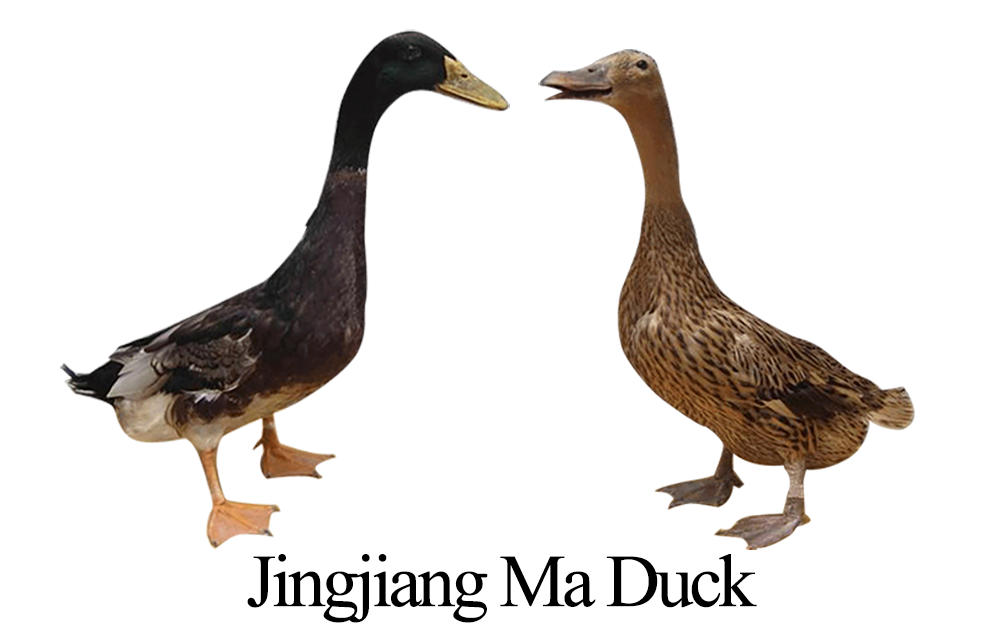

Supplement: Supplementary file 1 [file animals-13-01156-s001.zip › Figure S1/Jingjiang Ma Duck.tif]

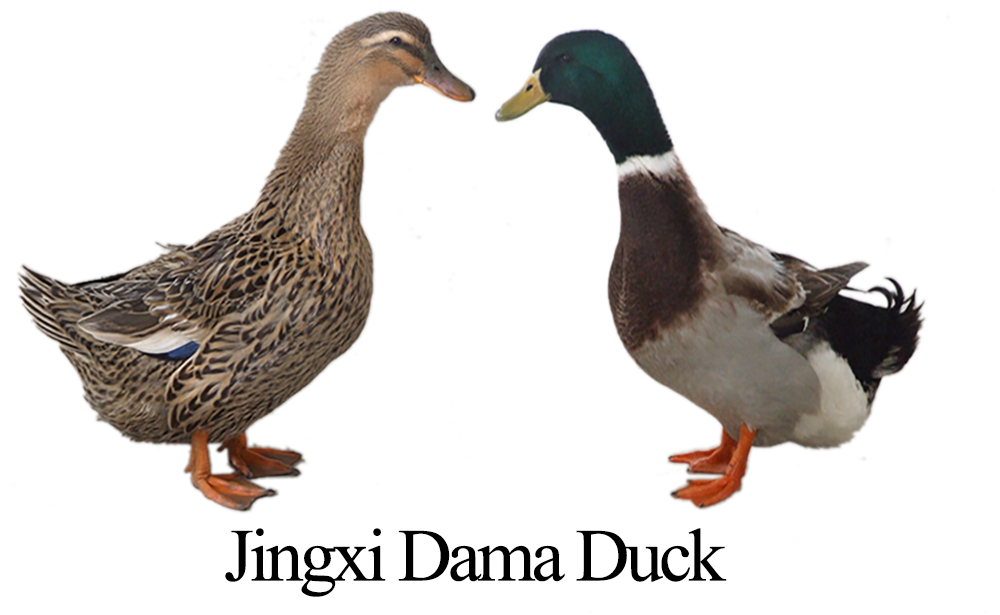

Supplement: Supplementary file 1 [file animals-13-01156-s001.zip › Figure S1/Jingxi Dama Duck.tif]

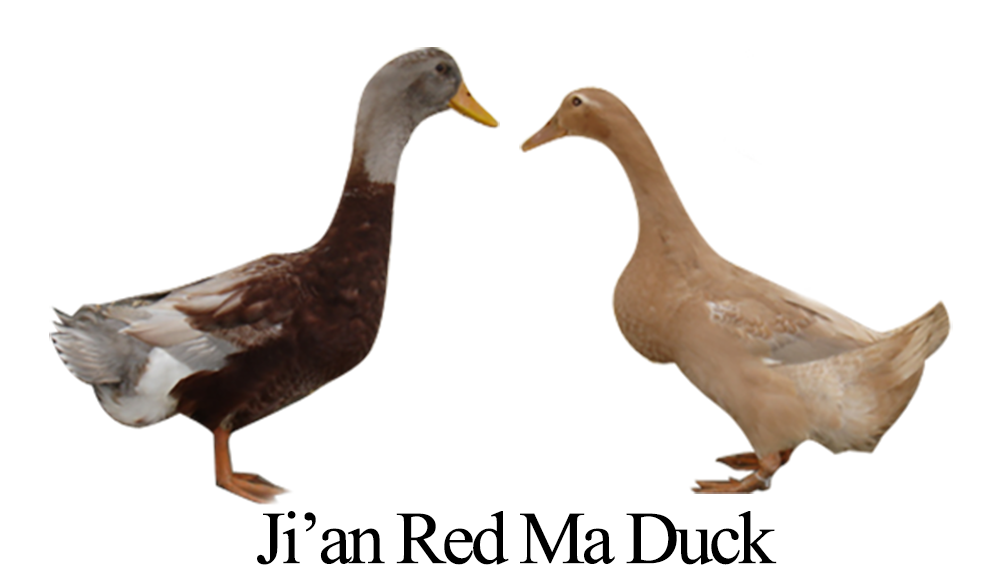

Supplement: Supplementary file 1 [file animals-13-01156-s001.zip › Figure S1/Jií»an Red Ma Duck.tif]

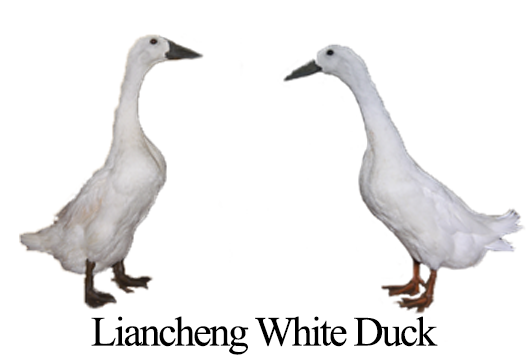

Supplement: Supplementary file 1 [file animals-13-01156-s001.zip › Figure S1/Liancheng White Duck.tif]

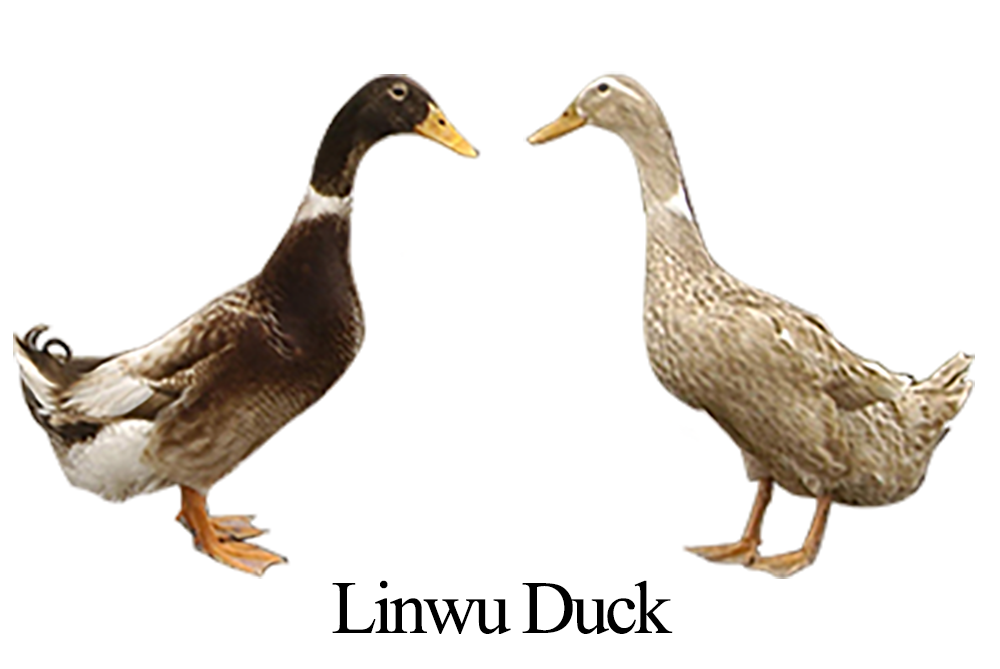

Supplement: Supplementary file 1 [file animals-13-01156-s001.zip › Figure S1/Linwu Duck.tif]

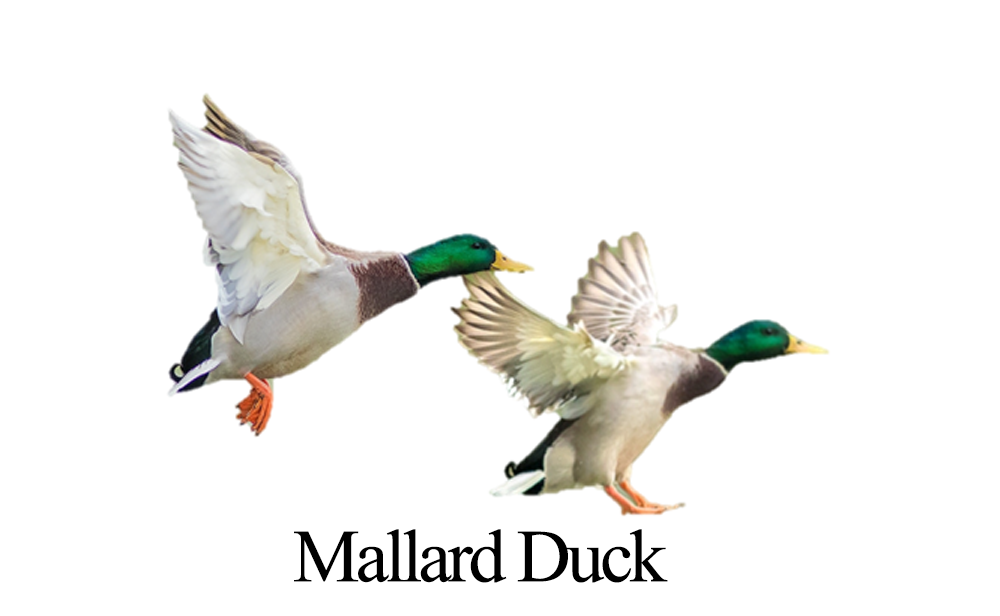

Supplement: Supplementary file 1 [file animals-13-01156-s001.zip › Figure S1/Mallard Duck.tif]

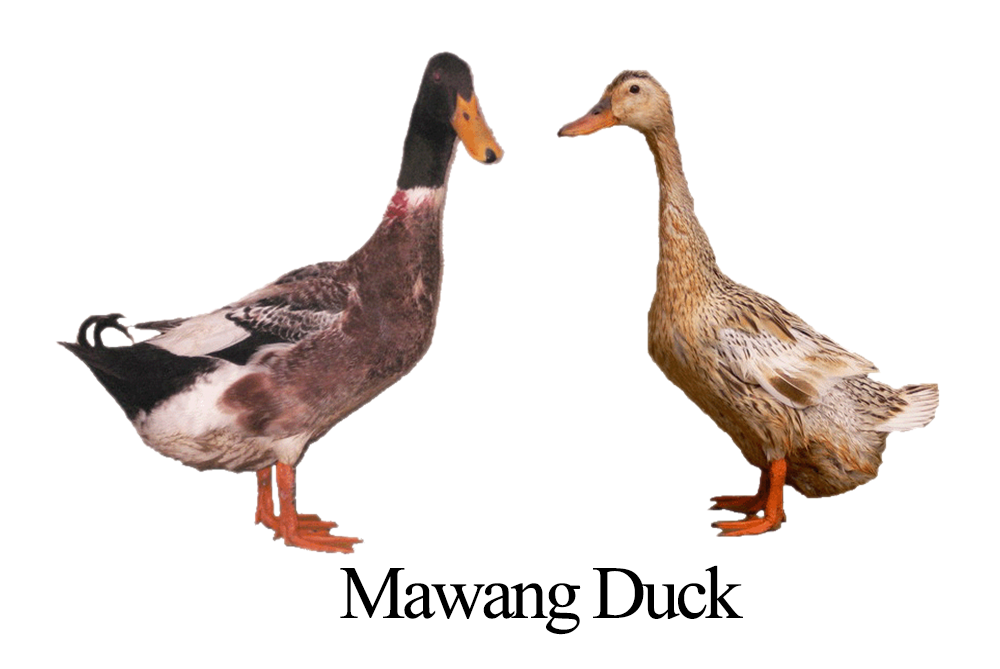

Supplement: Supplementary file 1 [file animals-13-01156-s001.zip › Figure S1/Mawang Duck.tif]

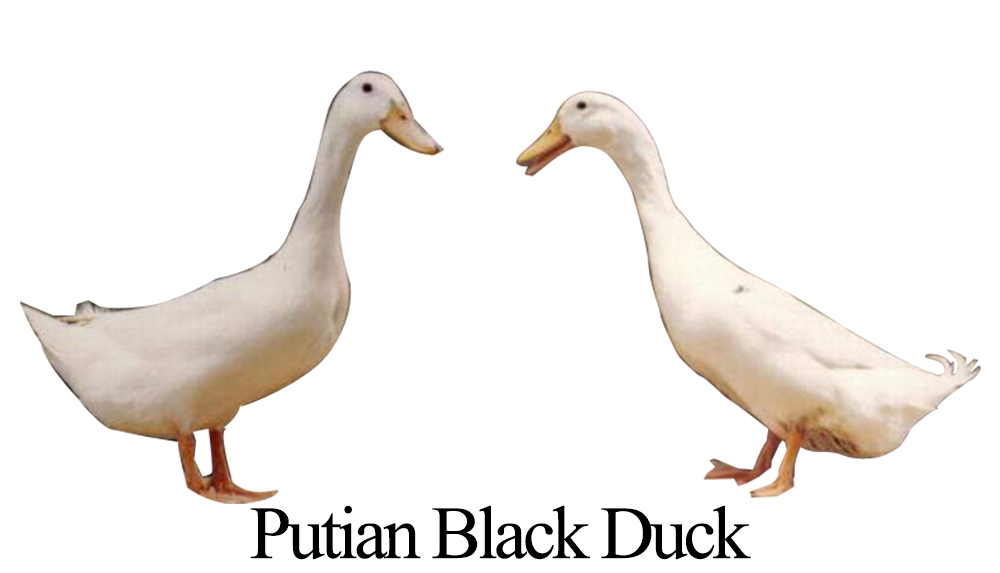

Supplement: Supplementary file 1 [file animals-13-01156-s001.zip › Figure S1/Putian Black Duck.tif]

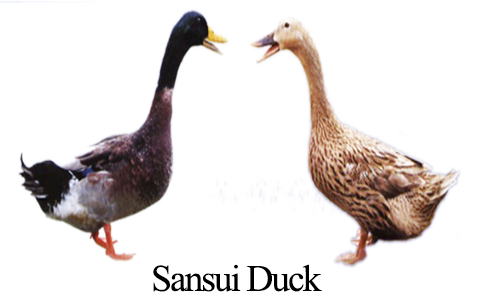

Supplement: Supplementary file 1 [file animals-13-01156-s001.zip › Figure S1/Sansui Duck.tif]

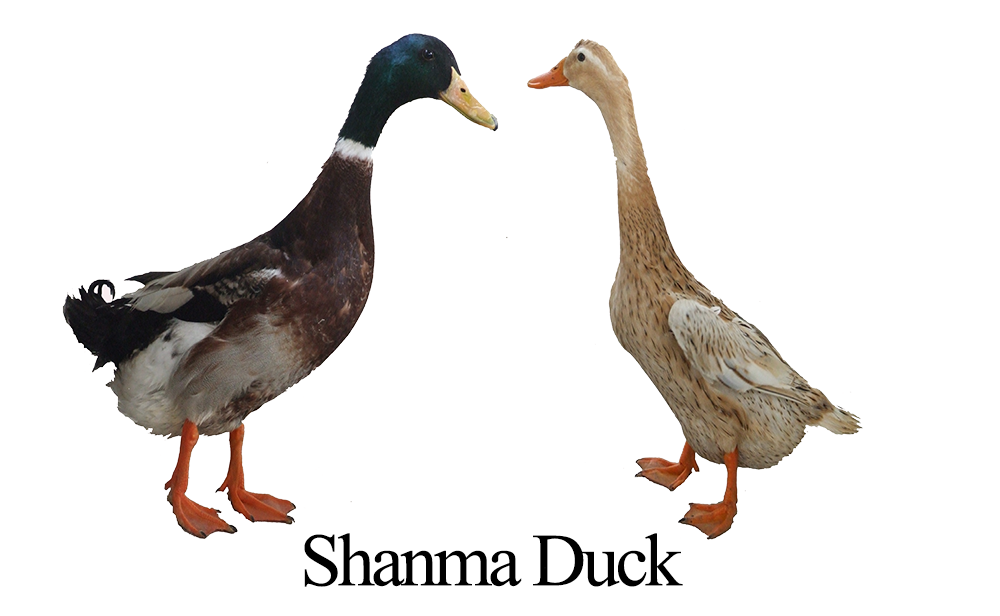

Supplement: Supplementary file 1 [file animals-13-01156-s001.zip › Figure S1/Shanma Duck.tif]

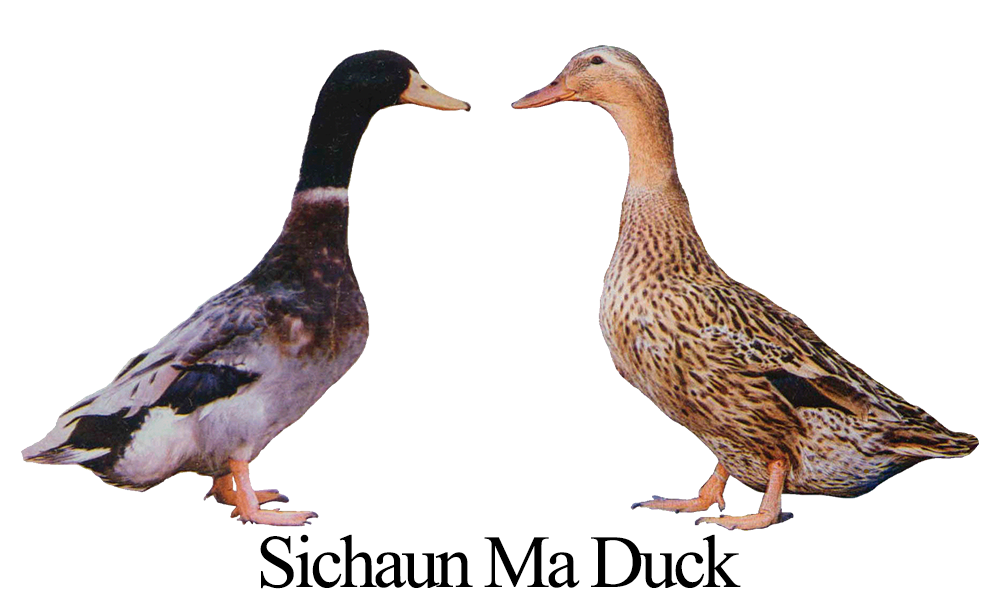

Supplement: Supplementary file 1 [file animals-13-01156-s001.zip › Figure S1/Sichuan Ma Duck.tif]

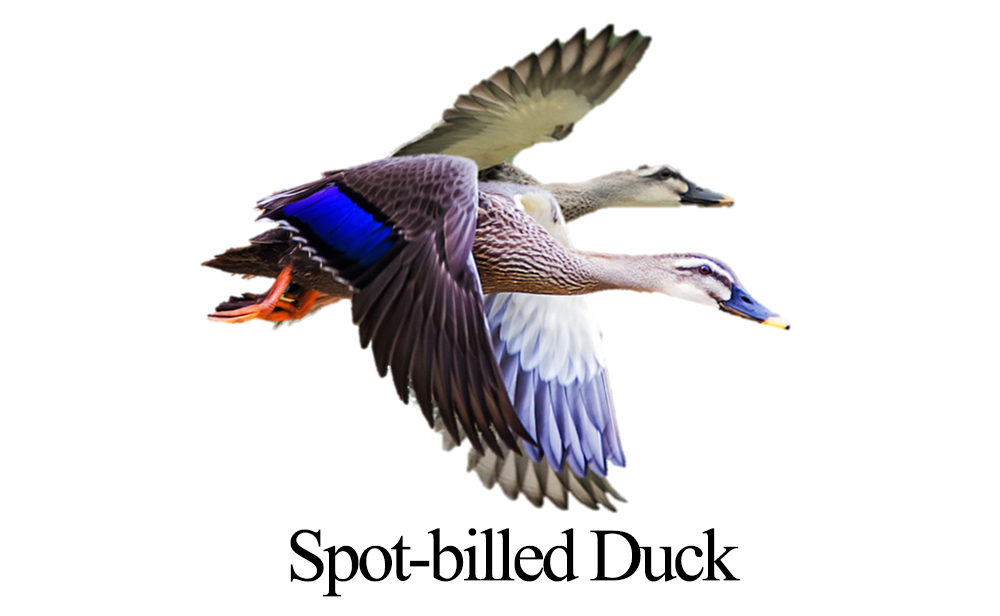

Supplement: Supplementary file 1 [file animals-13-01156-s001.zip › Figure S1/Spot-billed Duck.tif]

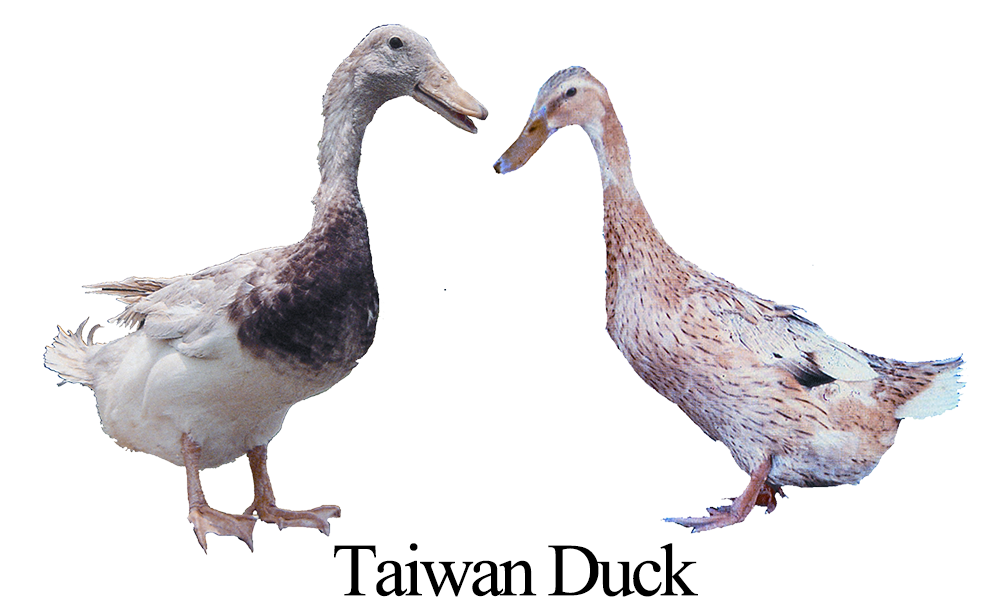

Supplement: Supplementary file 1 [file animals-13-01156-s001.zip › Figure S1/Taiwan Duck.tif]

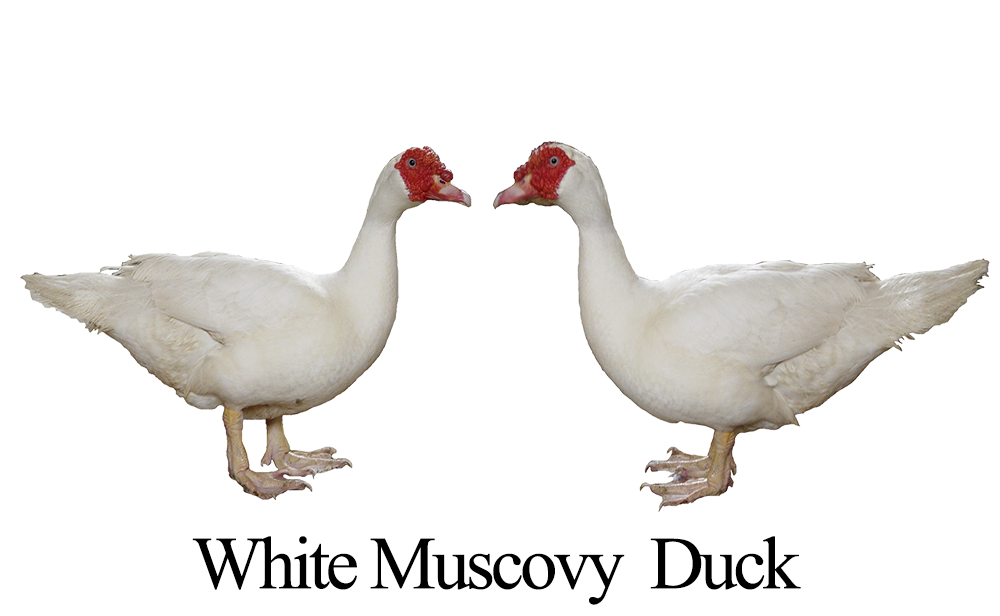

Supplement: Supplementary file 1 [file animals-13-01156-s001.zip › Figure S1/White Muscovy Duck.tif]

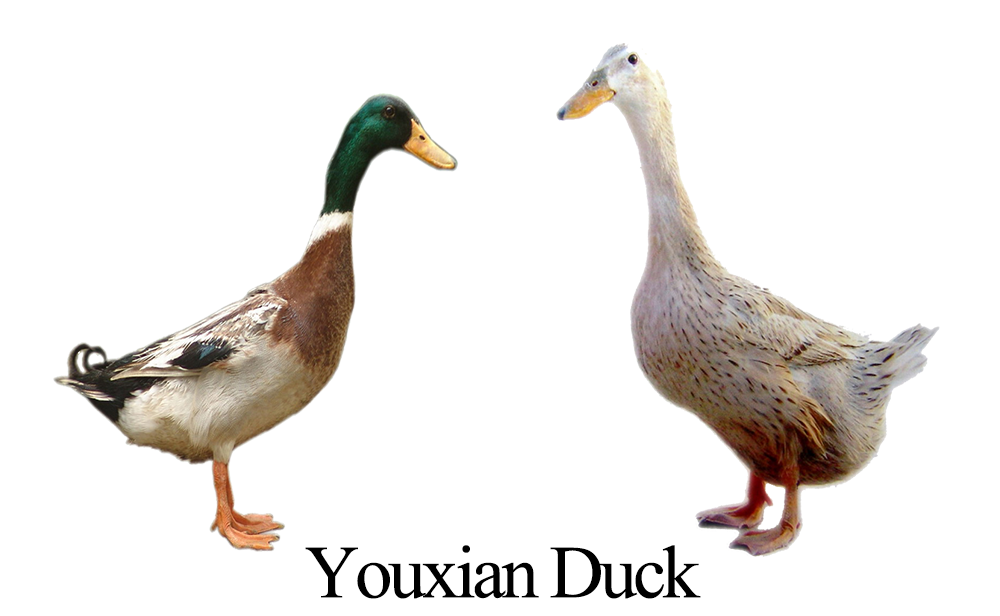

Supplement: Supplementary file 1 [file animals-13-01156-s001.zip › Figure S1/Youxian Duck.tif]

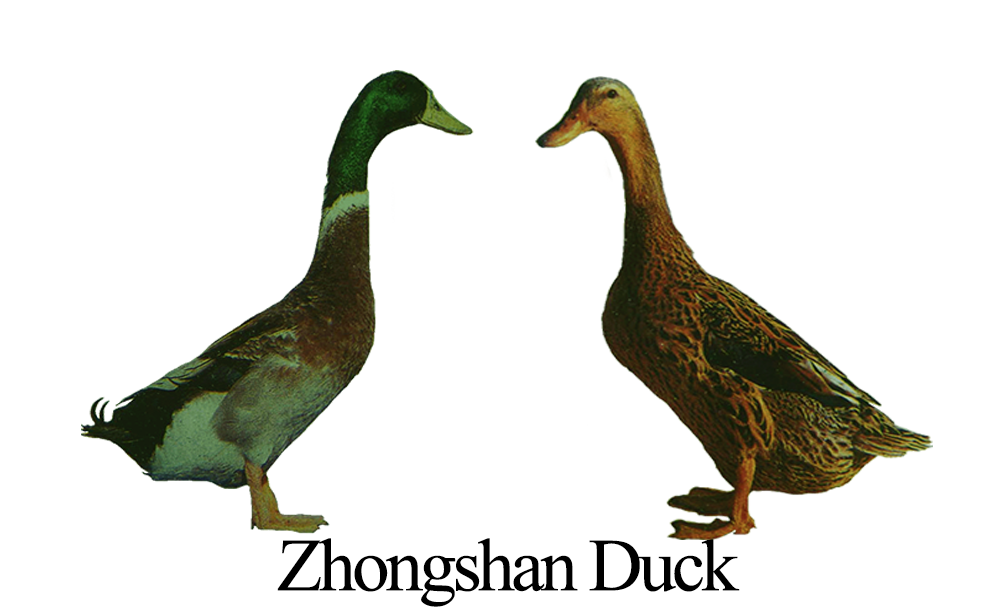

Supplement: Supplementary file 1 [file animals-13-01156-s001.zip › Figure S1/Zhongshan Duck.tif]

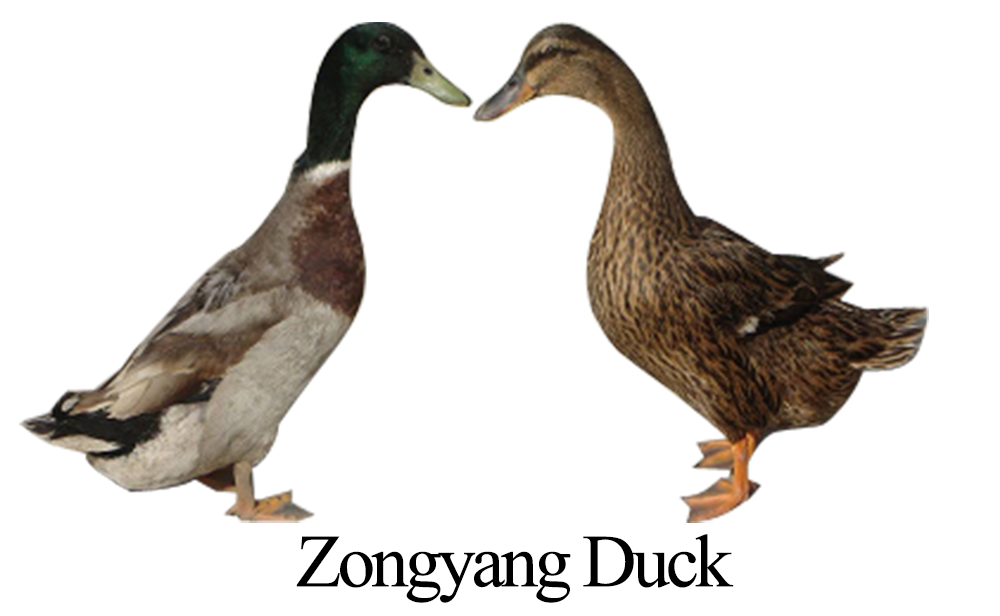

Supplement: Supplementary file 1 [file animals-13-01156-s001.zip › Figure S1/Zongyang Duck.tif]
